# Systems thinking in combating infectious diseases

**DOI:** 10.1186/s40249-017-0339-6

**Published:** 2017-09-11

**Authors:** Shang Xia, Xiao-Nong Zhou, Jiming Liu

**Affiliations:** 1 National Institute of Parasitic Diseases, Chinese Center for Disease Control and Prevention, Shanghai, 200025 People’s Republic of China; 2 Key Laboratory of Parasite and Vector Biology, National Health and Family Planning Commission, Shanghai, 200025 People’s Republic of China; 3 WHO Collaborating Centre for Tropical Diseases, Shanghai, 200025 People’s Republic of China; 4 Department of Computer Science, Hong Kong Baptist University, Kowloon Tong, Hong Kong; 5 CDC-NIPD & HKBU-CSD Joint Research Laboratory for Intelligent Disease Surveillance and Control, Shanghai, 200025 People’s Republic of China

**Keywords:** Systems thinking, Complex systems approach, Infectious disease control

## Abstract

The transmission of infectious diseases is a dynamic process determined by multiple factors originating from disease pathogens and/or parasites, vector species, and human populations. These factors interact with each other and demonstrate the intrinsic mechanisms of the disease transmission temporally, spatially, and socially. In this article, we provide a comprehensive perspective, named as systems thinking, for investigating disease dynamics and associated impact factors, by means of emphasizing the entirety of a system’s components and the complexity of their interrelated behaviors. We further develop the general steps for performing systems approach to tackling infectious diseases in the real-world settings, so as to expand our abilities to understand, predict, and mitigate infectious diseases.

**Electronic supplementary material:**

The online version of this article (doi:10.1186/s40249-017-0339-6) contains supplementary material, which is available to authorized users.

## Multilingual abstracts

Please see Additional file 1 for translation of the abstract into the six official working languages of the United Nations.

## Background

According to *A Dictionary of Epidemiology*, epidemiology in general deals with “the study of the occurrence and distribution of health-related states or events in specified populations, including the study of the determinants influencing such states, and the application of this knowledge to control the health problems” [1]. In this regard, epidemiological studies in combating infectious diseases mainly focus on addressing the challenges from the following three aspects: (1) investigating tempo-spatial patterns of disease occurrence; (2) identifying and evaluating associated impact factors; (3) exploring and conducting effective intervention measures. In doing so, epidemiologists will make use different methods in data collection and analysis [2, 3]. On one hand, empirical methods are often used in the phase of disease surveillance, which is to collect and analyze observational data about disease occurrences descriptively (e.g., when, where, and who). The results of this phase will help identify the tempo-spatial patterns of disease occurrences in humans as well as discover the variations with reference to their social and demographical characteristics (i.e., age, gender, and ethnicity) [4]. Experimental methods are needed in field investigation so as to test epidemiological hypotheses that are relating the proposed causes to the observed effects, the findings of which may serve as the foundation for developing and conducting intervention measures [5]. On the other hand, theoretical methods are essential for the purpose of formally understanding and characterizing the causality of disease transmission as well as evaluating the effectiveness of interventions by means of establishing associative or causal relationships between impact factors and disease occurrences [6]. Mathematical and computational models (e.g., compartmental Susceptible-Infectious-Recovered (SIR) modeling and multi-agent modeling) together with scenario-based simulations are developed as predictive tools for characterizing the dynamics of disease transmission and evaluating interrelationships with various impact factors [7, 8].

**Fig. 1:**
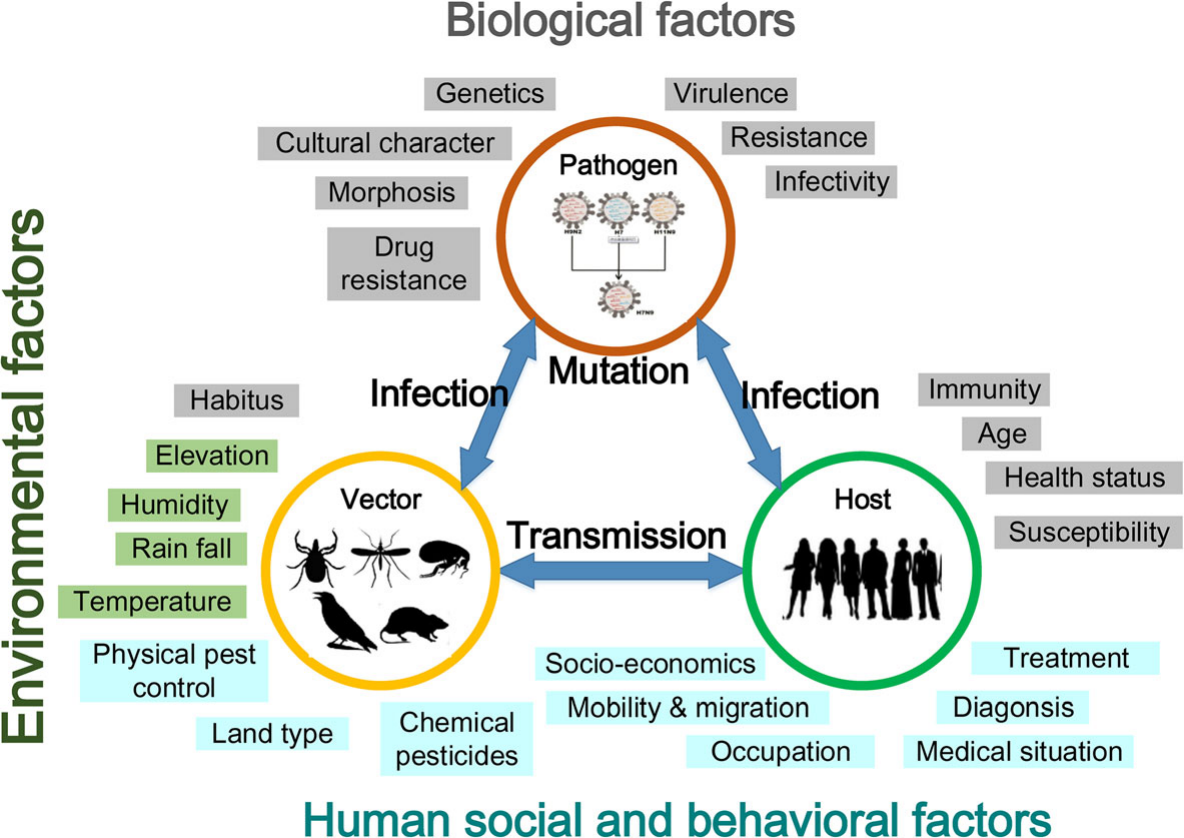
The basic interacting components (i.e., three circles) and multiple factors (i.e., shaded factors surrounding the components) affecting the transmission of infectious diseases

The existing methods have thrived for several decades and made great contributions in understanding and combating infectious diseases. However, there remain a number of challenges [9–11]. As schematically shown in Fig. 1, these challenges come from emerging and re-emerging infectious diseases, which are significantly correlated with the multiple impact factors and their interacting effects, including disease pathogen/parasite microbial genetic mutation, human socio-economic and behavioral changes, as well as environmental and ecological conditions. These interacting and coupling relationships among multiple impact factors have demonstrated the intrinsic mechanisms of the disease transmission temporally, spatially, and socially, while exhibiting systems characteristics of feedback, saturation, bifurcation, and chaos, etc., which make it hardly possible to utilize the conventional methods for comprehensive epidemiological investigations [12]. At the same time, the effective intervention measures rely on biomedical understandings of disease pathogens/parasites, descriptive studies on tempo-spatial patterns of disease occurrences, and casual analysis of impact factors. Besides, predictive explorations on the trends of disease transmission by exploring the mechanism-based interactions among the constituting components of disease transmission also play an important role in understanding and combating infectious diseases. For example, the early warning system for a newly emerging infectious disease, like H1N1 influenza, requires the knowledge about the possible geographical routes of disease transmission, such as human air-travel networks [13, 14]. The prevention of zoonotic and vector-borne diseases, like malaria, needs to address both environmental and ecological changes for vector species [15, 16] and human behaviors [17, 18], such as the migrant and mobile populations [19]. And furthermore, the effectiveness of conducted disease interventions depends on the efficacy of resource allocation, compliance of targeted host populations, and responsive feedback of environmental modifications.

In addition to the above-mentioned challenges, epidemiological studies also face with new opportunities in the presence of data-centric era, which is being enabled by the confluence of data from various sources and the development of modeling and analytical tools in data science. For example, WHO’s global disease surveillance system connects the health agencies of its member countries and partners at different scales, including local, regional, national, and international organizations. Such a surveillance system can be used for managing and sharing both historical records and reports on when and where some people have been infected by certain kinds of disease. In addition to those for disease surveillance and monitoring, other data sources are also helpful for analyzing and modeling potential disease transmission risks. For example, remote sensing data from satellites can readily be utilized in mapping the meteorological and ecological conditions of local or global environments, especially those remote and harsh regions, where field studies are either impossible or too costly to conduct due to their physical and political constraints [20, 21]. Another important source of data is from Internet-based media, which can serve as an informative channel for revealing individuals’ health related behaviors and opinions [22]. For example, Google flu trends was earlier used to assess the transmission of influenza virus [23, 24], and the use of Internet search data was demonstrated to be effective in predicting dengue [25].

In view of these challenges and opportunities, it would be imperative to develop new methods and paradigms that can offer a novel comprehensive perspective for investigating disease dynamics and associated impact factors, so as to expand our abilities to understand, predict, and mitigate infectious diseases.

## Discussion

### Systems thinking in combating infectious diseases

Systems thinking is a philosophical as well as methodological perspective that draws on the fundamental notions of systems theory that views a system as an integration of components together with the interacting relationships among them and with their residing environments [26–28]. Systems thinking emphasizes two fundamental concepts, i.e., complexity and entirety. Systems complexity is generated from the structure of integrated components, which is how the constituting components are organized and interact with each other and with the environments. Systems entirety is derived from the dynamic behaviors of a system as a whole, which is to say how a complex system of interacting components behaves and exhibits the emergent properties at the system level rather than a simple behavioral aggregation of its basic components.

Systems thinking offers a novel comprehensive perspective that examines the process of infectious disease transmission as a system with its structural complexity and behavioral entirety. In such a system, the components include disease pathogens/parasites, vector species, human populations, and their natural, social, and behavioral environments. The interactions among components are present, such as disease pathogen/parasite can infect and be transmitted between and/or within vector species and human populations. The interacting relationships of components with their relying environments can be described as components’ responses to the potential environmental changes, for example, biomedical genetic mutations of pathogens or parasites as a result of drug resistance selection, vector population fluctuations due to climate changes, and human exposure behavioral changes due to their socio-economic conditions. The emergent behaviors of such a system, i.e., the emerging and re-emerging infectious diseases, depend on the integrated effects of all the constituting components, including microbial evolutionary pathogens, zoonotic vector exposures, environmental changes, and human behavioral modifications.

Based on the above mentioned perspective of systems thinking, the studies of infectious diseases will go beyond the conventional methods that are usually confined by their disciplinary boundaries, such as the statistical analysis of disease occurrences or laboratory research on disease pathogens. The intervention measures in combating infectious diseases will be designed to modify the emergent behaviors at the system level, by exploring the interdisciplinary methods that aim to address systems complexity. Towards this end, we need a set of novel modeling and analyzing tools drawing on the concepts of systems thinking. This will enable the epidemiologists to develop and deploy more effective intervention measures.

### Complex systems approach in principle

The complex systems approach is a holistic approach that is intended to model, characterize, explain, and predict the emergent behaviors of a system with reference to its constituting complexity, which is hard to derive or compute by using conventional top-down reductionist approaches [29–31]. Such an approach pays a special attention to achieving the following three objectives [32, 33]:

1. **Systems modeling**
  The step of systems modeling provides a blueprint/framework that is abstracted and replicated from the real-world observations in the languages of mathematical/computational characterizations [7, 8, 34, 35]. Employing such a modeling analogy, therefore, requires identification, abstraction, and/or reproduction of certain observations, which is the starting point for the following steps of systems exploration and problem solving. For example, the compartmental models (e.g., the SIR model) for studying the influenza-like diseases use several compartments (i.e., susceptible, infectious, and recovered) to represent the different states of human infections (i.e., Susceptible-Infected-Recovered) [36]. In systems modeling, the basic components of a model also known as entities are the basic constituents of a complex system, which directly or indirectly interact among themselves as well as with their environments based on certain predefined or known mechanisms or principles. For example, in a network-based disease model, nodes represent human individuals whereas links represent the routes of disease transmission. Disease can be transmitted from one node to another due to the predefined contact interactions [37]. Interrelationships exist among entities and their local and global environments, through which the complex system as a whole exhibits its structural and behavioral complexity at and across various scales. Emergence, which is the dynamic transmission of infectious diseases, is defined in terms of the system-level patterns and regularities arising from the dynamics of a group of interacting entities, as generated from the reciprocally coupled and dynamically changed interrelations of the entities at multiple scales.
2. **Systems exploration**
  Systems exploration presents a set of analytical tools that are devoted to understanding the operating mechanisms underlying a complex system and, furthermore, finding explanations and making predictions about some observed systems’ dynamics. In order to uncover the operating mechanisms behind the observations, systems modeling will be performed to characterize or simulate the real system. For example, we can use the SIR model to characterize the dynamics of disease transmission in a human population. Then, by comparing the difference between the real-world observation and the synthetic simulation, the models and/or interaction mechanisms of the system will be fine-tuned, which may be reflected in the adjustments of the related model parameters, and the structure and behavior of the interacting entities. For example, when the incubation period is taken into consideration, the SIR model can be modified as the SEIR model with an additional latency compartment of “E” [8, 38].
3. **Problem solving**
  Problem solving emphasizes the ability of the complex systems approach to find its own way to achieve adaptive solutions that are well suited to the problem settings at hand. The ultimate goal is to develop a set of analytical algorithms that can adjust its own parameters for different application domains. For example, adaptive evolutionary algorithms can be used to automatically tune some of the parameters as related to the developed systems modeling or the proposed operating mechanisms. Constrained optimization algorithms are dedicated to finding the optimal solutions in resource allocation.

The complex systems approach can be used not only to build a modeling framework for mapping real-world observations/phenomena in analytical languages (i.e., mathematical and computational models), but also to reveal the operating mechanisms behind a complex system. Problem solving is the application of systems modeling and exploration with respect to the specific domain problem set in advance.

### Complex systems approach in practice

Generally speaking, in combating infectious diseases, the complex systems approach can help us understand how the systems of infectious diseases are organized in terms of the causal relationships and the impact factors on disease prevalence, how such systems behave over time and space by revealing the tempo-spatial distributions of disease occurrences, and how the diseases can be better mitigated and eradicated by developing more effective solutions for infectious disease control.

**Fig. 2:**
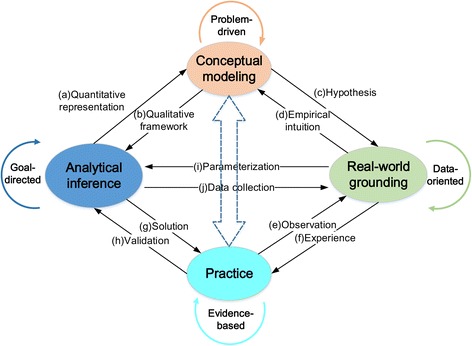
The four essential steps (in ovals) for performing the complex systems approach in combating infectious diseases. The directional arrows show their functional interrelationships

Specifically, Fig. 2 provides a schematic framework outlining the four essential steps for performing the complex systems approach to the epidemiological studies of infectious diseases.

- **Problem-driven conceptual modeling** first translates the real-world problems in an epidemiological domain into conceptual models in a theoretical or computational domain, which are aimed to describe the systems components in infectious disease transmission, their impact factors, and interaction relationships.
- **Data-oriented real-world grounding** then concentrates on discovering ways of embodiment of conceptual models, through the model parameterization, by means of obtaining and utilizing *real-world data* and/or statistic analysis of the real-world observations.
- **Goal-directed analytical inference** is devoted to further developing analytical methods and solutions in addressing specific real-world problems of disease surveillance and control, that is, to find right analytical methods and solutions to meet the specific goal*s*.
- **Evidence-based practice** proceeds to the implementation, validation, and improvement of the developed analytical solutions, aiming to bridge the theoretical and/or computational analysis with the real world.

Specifically, in the step of conceptual modeling, the goal is to build theoretical or computational prototypes of infectious disease systems, which can be used to represent the real-world problems. Based on the existing understanding/theoretical/empirical knowledge about infectious diseases and the related impact factors, mathematical and computational models can be used as a conceptual framework to reproduce the dynamics of infectious diseases. For example, in the case of influenza, the demographical profiles and contact structure of a human host population can be used to model disease transmission among different human groups. In the case of malaria, environmental factors, such as rainfall and temperature, can be identified from various sources, which provide us a causality analytical model for examining the population development of disease vectors. In order to achieve the above, we need to perform model selection with reference to the specific characteristics of the epidemiological problems at hand. For example, the compartmental models are well suited to characterizing disease dynamics in several host populations, such as in the case of influenza. On the other hand, the network models or agent-based models are more suitable for representing disease diffusion due to human movement behaviors, such as the imported malaria cases in the remote or cross-border areas [19]. As can be noted, conceptual modeling depends on simplifications and abstractions about the operating mechanisms of infectious diseases, which also set up hypotheses for the data collection in the step of real-world grounding (i.e., function c), such as the studies of influenza require the human social-economic data and human behavioral data, e.g., human air-traveling, and the studies of malaria require to collect the environmental data, such as rainfall and temperature. This step also provides a theoretical or computational qualitative framework for performing analytical inference methods (i.e., function b).

The goal of real-world grounding is to collect data from multiple sources and analyze such available data from different disciplines, aiming at a more comprehensive understanding about the structural interrelationships and behavioral mechanisms of real-world infectious disease systems. For example, the international airlines provide indirect networks for the transmission of H1N1 influenza worldwide [23]. The step of real-world grounding performs multi-disciplinary data fusion and knowledge discovery from massively accumulated data. The products of the data-oriented real-world grounding can in turn be used to provide empirical intuitions for conceptual modeling (i.e., function d), generate certain experience-based rules or principals to guide the practical implementation of infectious disease control measures (i.e., function f), and parameterize variables in performing inference algorithms (i.e., function i).

Based on the developed models and collected data, the step of analytical inference is to provide a series of specific problem-solving methods and solutions, which can be used as analytical tools for addressing the real-world problems that are taken into account in the step of conceptual modeling. For example, based on a network model, inference methods can be used to reveal the hidden pathways of malaria transmission in the remote or cross-border areas [39]. The gaps between the desired situations (goals) and the current situations (status quo) in disease surveillance and control will lead to the inference methods that lead to an improved solution. Performing analytical inferences will provide a set of quantitative representations for conceptual modeling (function a). For example, the inferred weights of network links denote the possibilities of malaria transmission among villages. Furthermore, the end products of this step can also develop solutions for the practical realization of infectious disease control (function g) and guide the data collection in the step of real-world grounding (function j). For example, ranking algorithms can help identify the relative risks of malaria for various villages in the remote or cross-border areas. At the same time, as more data are accumulated, the results of risk ranking will become more precise and reliable.

The fourth step of evidence-based practice concerns the application and validation of the developed solutions in the real-world practice of infectious disease surveillance and control. The goal of this step is twofold: (1) guiding the practice of disease control and prevention (function e); (2) validating and improving the applied analytical methods (function h). For example, active surveillance planning methods can help public health authorities decide how to distribute their very sparse resources to high-priority regions, so as to maximize the outcomes of disease intervention. The feedback from the field practice will help validate the analytical results and determine if the selected models and adopted inference methods can represent the real-world scenario and thus address the real-world problems. In other words, theoretical analysis and results will be used to guide the practice of infectious disease control, which will in turn validate or improve the developed models and inference methods.

## Conclusions

Systems thinking aims to better understand and characterize the complexity involved in the process of disease transmission and the implementation of intervention measures. A complex systems approach emphasizes the importance of the “holistic” context. The application of the complex systems approach in the specific context of epidemiology provides us a set of analytical tools to characterize the structure and impact factors of systems components, to capture the dynamics of how they interact with each other, and to evaluate and further improve the disease intervention measures. Systems thinking together with the complex systems approach represents a new era in epidemiological studies, which offers a comprehensive perspective for epidemiology (conceptual modeling, data grounding, analytical inference, and intervention practice), while integrating data from a wide range of sources and utilizing methods from diverse disciplines.

## Additional file

Additional file 1: Multilingual abstracts in the six official working languages of the United Nations. (PDF 497 kb)

## Abbreviations

SEIR: Susceptible-Exposed-Infectious-Recovered
SIR: Susceptible-Infectious-Recovered
WHO: World Health Organization
