# Supplementary material for: Systems thinking in combating infectious diseases
Source: Infect Dis Poverty. 2017 Sep 11;6:144. doi: 10.1186/s40249-017-0339-6 (PMC5594605; doi:10.1186/s40249-017-0339-6)
Supplement: Additional file 1: — Multilingual abstracts in the six official working languages of the United Nations. (PDF 497 kb) [file 40249_2017_339_MOESM1_ESM.pdf]

Translation of the abstract into the six official working languages of the United Nations

النظم الفكرية في مكافحه الامراض المعدية

شانغ شيا ، شياو نونغ تشو ، جيمنج ليو

الملخص
يعتبر إنتقال الامراض المعدية عمليه ديناميكية تحددها عوامل متعددة ناشئة من مسببات الأمراض أو الطفيليات وأنواع النواقل والمجموعات البشرية وتتفاعل هذه العوامل مع بعضها البعض لتوضح الأليات الجوهرية لانتقال المرض من الناحية الزمانية والمكانية والاجتماعية وفي هذه المادة العلمية الحالية نقدم منظورا شاملا يسمي بالنظم الفكرية للتحقيق في ديناميكيات الامراض وعوامل المؤثرة المرتبطة بها وذلك بالتشديد على مكونات النظام بأكملها وعلى تعقيد سلوكياتها المترابطة كما اننا نطور الخطوات العامة لتنفيذ نهج النظم لمعالجه الامراض المعدية وفق معطيات الواقع وذلك لتوسيع قدراتنا على فهم الامراض المعدية والتنبؤ بها والتخفيف من حدتها.

Translated from English version into Arabic by Mohamed R. Habib

系统论思想与传染病防控研究

夏尚, 周晓农, 刘际明

摘要
传染病传播扩散的动态过程受到多种因素的影响，如致病病原体或寄生虫、媒介生物和易感人群等。这些影响因素伴随疾病的传播扩散在时间、空间以及社会等多维度上互相关联、耦合和作用。本文阐述了如何利用系统论思想研究疾病传播扩散动力学过程及其相关影响因素的关联耦合作用。基于系统论思想，强调了疾病传播扩散中各个组成部分由于共同作用而表现出的系统整体性，以及由于交互耦合而表现出的系统复杂性。本文进一步归纳了在传染病研究中应用复杂系统理论方法的一般步骤，以期为传染病的防治、监测和消除提供新的科学视角。

Translated from English version into Chinese by Shang Xia

L’approche par systèmes de la lutte contre les maladies infectieuses

Shang Xia, Xiao-Nong Zhou, Jiming Liu

Résumé
La transmission des maladies infectieuses est un processus dynamique déterminé par de nombreux facteurs liés aux pathogènes et/ou parasites, aux espèces vectrices et aux populations humaines. Ces facteurs sont en interaction et présentent des mécanismes intrinsèques de transmission de la maladie dans le temps, l’espace et la société. Nous présentons ici un point de vue global, appelé « approche par systèmes », pour l’étude de la dynamique des maladies et des facteurs d’influence associés, au moyen d’une approche d’ensemble des composants du système et de la complexité de leurs comportements interdépendants. Nous développons en outre les grandes étapes de l’approche par systèmes appliquée à la lutte contre les maladies infectieuses dans les conditions réelles, dans le but d’élargir nos capacités à comprendre, prédire et freiner les maladies infectieuses.

Translated from English version into French by Suzanne Assenat, through

Системный подход в борьбе с инфекционными заболеваниями

Шан Ся, Сяо-Нун Чжоу, Цзимин Лю

Аннотация
Передача инфекционных заболеваний представляет собой динамический процесс, который формируется под воздействием многих факторов, создаваемых возбудителями болезней и/или паразитами, видами переносчиков болезней и народонаселением. Эти факторы взаимодействуют друг с другом и демонстрируют внутренние механизмы передачи болезней во времени, пространстве и обществе. В этой статье мы представляем всесторонний подход к изучению динамики заболеваний и воздействия связанных с этим факторов, получивший название "системное мышление", и обращаем внимание на важность рассмотрения в совокупности всех компонентов системы и сложности их поведения во взаимосвязанных системах. Мы далее развиваем общие шаги для применения системного подхода к борьбе с инфекционными заболеваниями в реальной жизни с тем, чтобы расширить наши возможности понимать, прогнозировать и снижать распространение этих болезней.

Translated from English version into Russian by Natalia Potashnik, through

Pensamiento sistémico para combatir las enfermedades infecciosas

Shang Xia, Nong Xiao Zhou, Jiming Liu

Resumen
La transmisión de las enfermedades infecciosas es un proceso dinámico determinado por múltiples factores procedentes de patógenos o parásitos, especies vectores y poblaciones humanas. Estos factores interactúan entre si y muestran los mecanismos intrínsecos de la transmisión de enfermedades de forma temporal, espacial y social. En este artículo, proporcionamos una perspectiva integral, denominada pensamiento sistémico, para investigar la dinámica de la enfermedad y los factores de impacto asociados, haciendo hincapié en la totalidad de los componentes del sistema y la complejidad de sus comportamientos interrelacionados. Además

desarrollamos los pasos generales para realizar el enfoque de sistemas y hacer frente a las enfermedades infecciosas en situaciones reales, con el fin de ampliar nuestra habilidad para comprender, predecir y mitigar las enfermedades infecciosas.

Translated from English version into Spanish by Aitana Pascual, through

TRANSLATORS
WITHOUT BORDERS
